# Early incorporation of body capital in adolescent beauty culture: A qualitative study of the ‘Sephora Kids’ phenomenon

**DOI:** 10.1371/journal.pone.0356694

**Published:** 2026-09-16

**Authors:** Monika Dobrogowska

**Affiliations:** Department of Sociology of Culture, Religion and Social Participation, John Paul II Catholic University of Lublin, Lublin, Poland; Xi'an University of Posts and Telecommunications, CHINA

## Abstract

**Objective:**

Adolescence is a developmental stage in which identity formation, bodily self-awareness, and social evaluation intensify, increasingly within digital and consumer environments. In recent years, growing attention has been directed toward the so-called “Sephora Kids” phenomenon, referring to children and early adolescents who engage in advanced skincare routines, use premium cosmetic products, and emulate adult beauty influencers through social media platforms. Despite its visibility in media discourse, empirical research on this phenomenon remains limited. This study explores how adolescents themselves understand skincare practices, beauty consumption, and age-related boundaries associated with the “Sephora Kids” phenomenon.

**Methods:**

The analysis is based on 30 in-depth interviews conducted with adolescents aged 13–16 in Poland. A qualitative research design and reflexive thematic analysis were employed to capture participants’ everyday routines, sources of beauty knowledge, peer and parental influences, and perceptions of health risks and age appropriateness.

**Results:**

The findings show that beauty practices are highly normalized in adolescents’ daily lives and are often organized as structured routines resembling adult cosmetic regimens. Social media platforms, particularly TikTok and Instagram, function as environments of informal learning, where algorithmically curated content and influencer practices shape both knowledge and expectations. Participants demonstrate considerable consumer literacy, including brand awareness and price sensitivity, while simultaneously expressing ambivalent attitudes toward younger children’s use of advanced skincare products. Parental mediation emerges as a significant factor, ranging from shared beauty practices to restrictive gatekeeping. Adolescents also articulate reflexive awareness of age-inappropriateness and potential health risks, often drawing on quasi-expert discourses circulating in digital and peer environments.

**Significance:**

By foregrounding young people’s perspectives, this study contributes to sociological debates on digital consumer culture, algorithmic socialisation, and the embodiment of aesthetic practices. The findings suggest that engagement in beauty routines can be understood as an early stage in the incorporation of body-related resources, interpreted here as emerging forms of body capital. In dialogue with existing research on aesthetic and cultural capital, the study demonstrates that such processes are not only socially distributed but also developmentally internalised through routine practices shaped by platform environments.

## Introduction

Adolescence is a developmental stage characterized by intensified identity work, heightened sensitivity to social evaluation, and increasing autonomy in everyday decision-making. In contemporary digital environments, these processes increasingly unfold through embodied practices, appearance-related choices, and everyday consumption activities. Social media platforms such as TikTok, Instagram, and YouTube expose young people to a constant stream of beauty-related content, including skincare advice, aesthetic norms, and commercial product recommendations. Digital visibility, peer feedback, and algorithmic amplification intertwine to position appearance-related practices as salient sites of self-evaluation and social belonging. Recent research demonstrates that digital platforms play a central role in shaping adolescents’ understandings of their bodies, self-presentation, and social value [1–3]. As a result, beauty routines have become embedded in broader cultural expectations surrounding self-care, discipline, and continuous self-optimization [4].

A particularly visible manifestation of these developments is the phenomenon commonly referred to as “Sephora Kids.” The term is generally understood to describe children and early adolescents belonging to Generation Alpha – those born after 2010 – who have grown up immersed in digital environments from an early age [5]. The concept of “Generation Alpha” was introduced by Australian demographer Marc McCrindle [6], and some sources also attribute to him the popularization of the label “Sephora Kids” [7]. Amplified by international media since 2023–2024, the term refers to children and young adolescents who incorporate multi-step skincare routines, purchase high-potency cosmetic products, and emulate adult beauty influencers [8]. Reports from dermatologists increasingly highlight the use of retinol, exfoliating acids, and peptide-based products among preteens, despite potential risks to developing skin [9]. Viral online content frequently showcases 10–13-year-olds performing complex routines, discussing “barrier repair” or “anti-aging,” and recording “get ready with me” videos modeled on adult aesthetics [10]. Although often framed as a cultural curiosity or moral panic in public discourse, this phenomenon reflects deeper structural transformations in youth consumer culture, including accelerated consumer socialisation, the expansion of aesthetic labour into early adolescence, and the platformisation of bodily self-management [11,12].

Despite its visibility in media and public debate, the “Sephora Kids” phenomenon remains largely unexplored in academic research. Existing discussions appear mainly in journalistic commentary and social media debates [13], while peer-reviewed studies addressing children’s engagement with advanced skincare, anti-aging routines, or beauty influencer practices are virtually absent. While earlier qualitative research has examined adolescents’ attitudes toward skincare and beauty practices in digital contexts [14], no empirical studies to date have explored how adolescents themselves interpret the incorporation of these practices as part of broader processes of bodily valuation, boundary negotiation, and perceived risk. This gap indicates that the phenomenon is both emergent and theoretically underdeveloped, making it a timely and important subject for scientific inquiry.

Young people’s engagement with cosmetic practices can be understood through the lens of body and aesthetic capital. Following Bourdieu, Shilling, and Turner [15–18], the body constitutes a malleable project shaped by cultural norms, social institutions, and individual aspirations, where appearance functions as a socially and digitally mediated resource. Skincare and beauty routines thus represent techniques through which adolescents manage, optimise, and present their bodies, participating in processes of distinction in which taste, style, and bodily presentation operate as markers of social position and aspirational belonging [19–22]. Empirical and theoretical work on cultural and social valuation of physical appearance, including studies by Kuipers [23,24], Sarpila et al. [25] and Schneickert et al. [23], highlights the relevance of social differences and cultural factors in evaluating beauty. Recent research (Feijoo&Vizcaíno-Verdú; Demetriou et al., Anderson et al.) further develops the concept of social media aesthetic capital, showing how curated digital self-presentation translates into peer recognition and perceived social success under platform pressures [26–28]. These approaches underscore that adolescents’ early engagement with beauty routines constitutes a form of embodied capital acquired, refined, and displayed through both everyday practice and online visibility.

The second central axis concerns algorithmic and social media socialisation. Mediatization theory conceptualises the transformation of social and cultural processes through digital communication infrastructures [29,30]. In the context of “Sephora Kids”, platform algorithms, influencer economies, and parasocial relationships shape the norms, techniques, and timing of beauty practices, guiding adolescents’ attention toward specific products and routines. Adolescents often perceive influencers as authentic and trustworthy authorities, which mediates uptake of aesthetic knowledge, consumption decisions, and the internalisation of bodily norms [31–37]. These dynamics illustrate how platform-mediated socialisation interacts with body and aesthetic capital, reinforcing early incorporation of socially recognised beauty practices.

Finally, situating these developments within broader consumer culture highlights the interplay between bodily capital, digital mediation, and market logic. Scholars of childhood and adolescent consumption emphasise that young people increasingly function as sophisticated consumer subjects, targeted by industries promoting identity work, aspirational lifestyles, and enhanced desirability [19,37–44]. Within this framework, “Sephora Kids” exemplify how digital, social, and commercial pressures converge to shape early investments in body capital, aesthetic routines, and peer-mediated recognition.

Against this backdrop, the present study examines how adolescents aged 13–16 understand skincare, cosmetics, and the broader cultural meanings associated with the “Sephora Kids” phenomenon. It focuses on: (1) the early incorporation of body capital into everyday routines; (2) the role of digital platforms and algorithmic environments in shaping beauty knowledge; (3) parental involvement as co-producers of consumption practices; and (4) negotiations of age boundaries and perceived risks. By connecting aesthetic and social media–based capital with platform-driven socialisation, the study contributes to emerging international discussions on youth, digital consumer culture, and the moral and social dimensions of bodily self-care.

## Materials and methods

### Study design

This study employed a qualitative research design based on individual in-depth interviews (IDI) to explore how adolescents understand skincare practices, beauty consumption and the broader cultural dynamics associated with the “Sephora Kids” phenomenon. A qualitative approach was selected because the research questions concerned subjective meanings, lived experience, and socially embedded practices. IDI enabled detailed exploration of routines, interpretations, and motivations that are not accessible through quantitative approaches.

This approach is consistent with established principles of qualitative inquiry, which emphasize the exploration of meaning, context and lived experience in social research [45].

### Participants and recruitment

Participants were adolescents aged 13–16 residing in Poland. A total of 30 interviews were conducted (27 girls and 3 boys). The sample reflects the empirical reality of the phenomenon under study, in which engagement with intensive skincare and cosmetic practices is strongly feminised. Rather than treating gender as a controlled variable, it is understood here as structurally embedded in the social organisation of aesthetic labour and consumption practices (Gill & Elias).

Participants were recruited between 21 March and 18 April 2025 using a purposive and snowball sampling strategy, initiated through academic and institutional networks at the Centre for Social and Economic Analyses at the Institute of Social Sciences, John Paul II Catholic University of Lublin. Inclusion criteria were: (1) age between 13 and 16, (2) active use of at least one social media platform (e.g., TikTok, Instagram, YouTube), and (3) familiarity with skincare or beauty practices.

Sampling aimed to ensure variation in place of residence (rural, small towns, medium-sized cities, large urban centres) and intensity of digital engagement. Socioeconomic variation was indirectly captured through recruitment diversity and narrative indicators (e.g., price sensitivity, brand literacy, “dupe” practices, and access to premium retail spaces).

### Data collection

Data were collected in spring 2025. Interviews were conducted face-to-face or online, depending on participants’ availability and parental preferences. A semi-structured interview guide was used to ensure consistency while allowing participants to elaborate on emerging topics. The interview guide was developed by the lead researcher and consulted with experts from related disciplines (pedagogy and psychology) to ensure appropriateness for adolescent participants.

Semi-structured interviews are particularly suitable for exploring meanings and interpretations, as they combine structure with openness [46].

Interview questions covered:

(1) daily skincare and makeup routines;
(2) perceptions of beauty and appearance;
(3) sources of beauty knowledge (e.g., influencers, peers, parents);
(4) views on the “Sephora Kids” phenomenon;
(5) experiences with cosmetic products, including those marketed for adults.

Interviews lasted between 17 and 43 minutes and were audio-recorded with consent from both adolescents and their parents or legal guardians. All recordings were transcribed verbatim in Polish.

No systematic differences in thematic content were observed between online and face-to-face interviews; both modes generated comparable depth and richness of data.

### Ethical considerations

The research protocol was approved by the Ethics Committee for Research in Social Sciences at the John Paul II Catholic University of Lublin (approval number: P-38/DKE/NS/2025).

Participation was voluntary. Written informed consent was obtained from all participants. Because participants were minors, written consent was also obtained from parents or legal guardians prior to participation, and assent was obtained from the adolescents themselves.

All consent forms are securely stored in accordance with university data protection policies on institutional servers. Interviews were conducted anonymously, and no identifying information was collected.

All personal identifiers were removed during transcription. Participants were assigned pseudonymous codes (e.g., P01, P07), which are used in the Results section when quoting interview material. Participants could withdraw from the study at any time without consequences.

### Data analysis

Data were analysed using a thematic analysis approach, following established guidelines for identifying, analysing and reporting patterns within qualitative data [47].

The analytic strategy combined an inductive, data-driven coding process with subsequent theoretical interpretation, resulting in a hybrid approach that moved iteratively between empirical material and conceptual explanation.

In qualitative interviewing, new themes and meanings often emerge during data collection that cannot be fully anticipated in advance. For this reason, coding was grounded in the empirical material rather than pre-defined theoretical categories. Analytical notes (memos) were produced throughout the research process to document emerging codes, their meanings, and relationships between categories, ensuring conceptual clarity and consistency in interpretation.

The analysis proceeded through the following stages:

1. Familiarisation with the data through repeated reading of transcripts.
2. Initial coding conducted manually by the lead researcher. Codes captured meaningful segments related to beauty practices, social influences and perceptions of risk.
3. Development of preliminary categories based on recurring patterns across interviews.
4. Iterative refinement of categories into broader thematic clusters through constant comparison across transcripts.
5. Construction of final themes, guided by theoretical concepts from the sociology of the body, mediatization and consumer culture.
6. Representative quotations were selected to illustrate each theme.

Importantly, theoretical concepts did not pre-structure the coding process. Coding remained primarily inductive, while interpretive frameworks were introduced at later stages of analysis. This allowed for continuous movement between data and theory, strengthening the empirical grounding of conceptual claims.

Data collection and analysis partially overlapped, allowing insights from earlier interviews to inform subsequent data collection, including the refinement of interview questions and probes.

The coding process was iterative and involved regular discussions within the research team. These discussions functioned as structured peer debriefing, during which emerging codes and preliminary themes were critically reviewed, compared, and refined. In cases of interpretive divergence, final decisions were reached through iterative discussion and re-examination of the data.

In addition to transcript-based analysis, observational insights recorded during interviews were incorporated, including participants’ emotional reactions, hesitations, tone of voice, and non-verbal cues. These elements supported the identification of sensitive topics and levels of engagement, enriching the interpretive depth of the analysis.

Coding was conducted manually using printed transcripts and written annotations; no qualitative software was used.

The aim of the analysis was not only to identify recurring themes but also to explore relationships between categories and variations across participants’ accounts, including divergent or contradictory perspectives.

Data saturation was assessed iteratively during data collection and analysis. Interviews were analysed in parallel with data collection, allowing continuous evaluation of emerging codes. Saturation was considered reached when no new substantive themes emerged in subsequent interviews, and later data served to further elaborate existing categories rather than generate new ones [48].

### Reflexivity

The interviewer shared a similar cultural background with participants but did not belong to their age group. Reflexive notes were taken after each interview to document emerging impressions and potential biases. The research team acknowledges that adolescents’ interpretations of beauty practices are shaped by broader cultural discourses, and analysis was conducted with attention to avoiding adult-centric assumptions. The analysis was conducted with awareness of the researcher’s interpretive role in shaping coding decisions and thematic construction. Particular attention was paid to avoiding adult-centric assumptions when interpreting adolescents’ practices and meanings. Regular discussions within the research team supported critical reflection on emerging interpretations and contributed to minimising potential bias.

## Results

### Theme 1. Everyday cosmetic routines and the normalisation of beauty work

Across interviews, adolescents described skincare and makeup routines as highly structured, repetitive, and temporally organised practices that closely resemble adult cosmetic regimes. These routines were not framed as occasional experimentation but as a taken-for-granted element of everyday functioning. The detailed sequencing of products, attention to ingredients, and differentiation between morning and evening care suggest an early internalisation of adult beauty norms and self-care logics, in which appearance management becomes a routine responsibility rather than a form of play.

Importantly, while these practices are described by participants as ordinary and age-appropriate, analytically they may be interpreted as early forms of routinised aesthetic work, in which knowledge, discipline, and bodily self-management are gradually stabilised as everyday competencies rather than exceptional efforts [26,27].

#### Morning and evening routines.

Participants outlined consistent sequences of steps that they repeated daily, often using precise terminology related to cosmetic products, ingredients, and application techniques:

“At the beginning of the day, I wash my face with water. Then I put on a cream… oh, Cetaphil! […] Only after that do I apply my makeup. And when I notice stronger breakouts, I start doing a deeper skincare routine in the evening.” (Interview 3).

“Every morning I wipe my face with a toner… I use a mattifying cream or a niacinamide gel. In the evening I use something with an acid, silica and charcoal… then niacinamide and a moisturising cream.” (Interview 14).

Several participants explicitly framed these routines as an everyday norm shared by their peers:

“I think that interest [in cosmetics] is quite big, because at my age a lot of my peers wear makeup, just like me, and practically every day at school I’m wearing makeup.” (Interview 6).

Others described routines that combined repetition with individual aesthetic preferences and expressive elements:

“First of all, I apply concealer on specific spots where it’s needed, but it’s always the same places, so it’s not over the whole face. Then I do my eyes - first I put on black and then I play with my colours. For example, lately I’ve been using more red and also applying it under my eyes. I put white in the inner corners. I do eyeliner - sometimes longer, sometimes shorter. Then mascara, sometimes a bit of highlighter on the cheeks or nose. And that’s basically it.” (Interview 28).

In some cases, routines involved a particularly extensive number of products and clearly mirrored adult beauty practices:

“First I apply a toner. Then bronzing ampoules, a serum and SPF 50… foundation, bronzer, concealer, blush, two types of powder, highlighter, pencils, mascara and a setting spray.” (Interview 20).

“When it comes to skincare, obviously there are various moisturising creams and cleansing foams. Before that, I also do peels, at least once or twice a week. And when it comes to makeup itself, I use a cream under makeup, then a makeup base. It also depends on how much time I have and how I want to do my makeup, but sometimes I apply foundation, then concealer, possibly blush and bronzer - it mainly depends on how much time I have. I also use lash oil. I think this is mostly my everyday routine.” (Interview 16).

Although the scope and complexity of routines varied, participants consistently described these practices as normal, habitual, and age-appropriate. This indicates that intensive beauty work has become embedded in everyday adolescent life rather than being perceived as excessive or exceptional.

#### Peer exchange and validation.

Beauty routines were not developed in isolation but were actively negotiated within peer groups. Peer interactions functioned both as sites of emotional validation and as informal channels for exchanging expert-like knowledge about products, ingredients, and techniques.

“If someone gets oily eyelids, we tell each other. Or when someone’s makeup turns out nice or they manage to glue on lashes […] we compliment each other.” (Interview 17).

“A friend of mine, maybe a year older than me, recommended retinol to me, saying that it helps with acne. I tested it myself for a while, but I immediately got really bad marks, so I stopped. I also know that at the beginning you need to use a smaller amount of that acid and gradually increase it.” (Interview 18).

“I like it - I feel better right away when I’m wearing makeup, more confident.” (Interview 23).

In this way, peer groups operated as micro-communities of practice, reinforcing both the emotional and technical dimensions of everyday beauty work.

#### Boys and grooming.

Participants noted that grooming practices are increasingly present among boys, particularly in relation to hair care and basic skincare:

“A lot of boys take care of their hair… that’s like 80% of a boy’s appearance. […] They also use creams, mostly in the higher grades.” (Interview 3).

“They definitely use basic products like shampoo, but from what I know they also use various hair conditioners. Especially if they have a styled haircut and need to fix it in the morning and style it again.” (Interview 29).

These accounts suggest a gradual normalisation of basic grooming practices among boys, indicating that beauty-related self-care is becoming less exclusively feminised within adolescent cultures.

#### Price sensitivity and product substitutions.

Economic considerations shaped product choices for some respondents, revealing varying degrees of consumer literacy and negotiation between affordability, perceived quality, and parental approval:

“If there’s a cheaper dupe of something expensive, why buy the expensive one? The cheaper one works the same.” (Interview 27).

“If it’s a good cosmetic product that I know for sure won’t cause an allergic reaction and is really worth the money, then if my mum agrees, I buy it.” (Interview 1).

These narratives indicate that even everyday cosmetic decisions are embedded in broader negotiations involving economic rationality, bodily safety, and family norms. What is more, these routinised practices indicate not only the normalization of beauty work, but also the early incorporation of bodily skills, aesthetic knowledge and self-discipline that can be interpreted as emerging forms of body capital.

These accounts also suggest that aesthetic practices are embedded in class-inflected consumption logics, where economic constraints, parental approval, and product knowledge intersect in shaping access to and valuation of beauty routines [23,49].

### Theme 2. Social media influence and aspirational beauty norms

Participants consistently emphasised the role of digital platforms – particularly TikTok and Instagram – in shaping their knowledge, expectations, and everyday practices related to beauty. Social media functioned not only as an instructional resource providing practical advice on cosmetics and skincare, but also as a symbolic space in which beauty norms, age ideals, and aspirational lifestyles were continuously circulated and amplified.

#### Visibility of very young beauty users.

Across interviews, adolescents frequently referred to online content featuring much younger children engaging in advanced beauty practices. These observations were often accompanied by comments on accelerated maturity and age-inappropriate behaviour.

“Girls aged 8-10 […] they had a whole routine and looked much older. They used more products than I do.” (Interview 28).

“Very young girls, even in grades 1-3 [around 7-8 years old], already go to Sephora and behave as if they were adults.” (Interview 13).

These accounts indicate that exposure to very young beauty users online contributes to the normalisation of advanced cosmetic practices at increasingly younger ages, reinforcing perceptions that intensive beauty work is both expected and socially visible early in life.

#### Learning through online content.

Many adolescents described the internet as their primary source of beauty-related knowledge, emphasising visual platforms as key spaces for learning techniques, discovering products, and evaluating cosmetic effectiveness.

“I learned about makeup from the internet… videos, TV… I follow women on TikTok.” (Interview 21).

“If something pops up for me, I watch makeup recommendations.” (Interview 1).

Several participants highlighted how popularity on social media influenced their interest in specific products and encouraged further information-seeking:

“Recently I saw that some new concealer was popular on TikTok and that I would like to test it because a lot of people recommend it. […] I also look online for opinions from other people, whether it worked for them or not. […] I often search on TikTok.” (Interview 22).

Others described learning beauty routines through highly stylised celebrity content:

“There is a channel on YouTube called Vogue where different celebrities show their makeup routines. I like watching that, and if I like something, I use it later myself.” (Interview 25).

Together, these narratives suggest that digital platforms operate as informal educational environments in which adolescents acquire beauty knowledge through observation, imitation, and peer-like evaluation rather than through expert or institutional guidance.

### Influencer lifestyles as aspirational models

Influencers were frequently perceived as powerful reference points shaping both appearance ideals and broader lifestyle aspirations.

“Influencers have an impact… they show this cool life and kids want to live like that… But not everyone will get those free PR packages.” (Interview 4).

This reflects a wider process in which beauty practices become embedded in aspirational narratives linking appearance, consumption, and social success, while simultaneously obscuring the commercial structures underpinning influencer content.

#### Understanding the “Sephora Kids” phenomenon.

Participants demonstrated a shared and relatively coherent understanding of the “Sephora Kids” phenomenon, often linking it to age, consumer excess, and online visibility.

“These are children, around 10-13, who use expensive products not meant for their age and show off with them on TikTok.” (Interview 18).

Others emphasised the growing presence of children in premium beauty retail spaces:

“In Sephora and generally in different store chains where cosmetics are sold, children have also started buying things, kind of on a mass scale. I have witnessed a group of children entering Sephora and buying the most expensive cosmetics. It’s simply that small children start wearing makeup at a very young age.” (Interview 16).

Participants also described disruptive behaviours associated with these practices:

“These are very young girls, maybe 14 at most, but often younger, who walk around different drugstores and expensive shops with colourful cosmetics. They […] use the testers, put makeup on with them, and this often leads to the testers being destroyed. I don’t encounter this phenomenon very often myself, but it seems noticeable - if ‘those Sephora kids’ were somewhere, the stores look very specific afterwards.” (Interview 15).

These descriptions frame the phenomenon not only as a digital trend but also as a visible and material presence in offline consumer spaces, reinforcing perceptions of boundary-crossing and excess.

#### Algorithm-driven exposure.

Beauty-related content was often encountered passively through automated recommendations rather than through deliberate searching.

“Popular topics just appear for me… sometimes I watch, sometimes I skip.” (Interview 21).

“I follow some famous people, but mostly I learn about cosmetics on the internet when something just happens to appear for me randomly. I don’t follow any profiles specifically about that.” (Interview 18).

In several cases, algorithmic exposure translated directly into consumption-oriented conversations within the family:

“A cosmetic often shows up for me online and then I tell my mum that I would like to try or test it. And usually I buy it later.” (Interview 23).

These narratives suggest that algorithmic systems function as powerful, though largely invisible, agents of socialisation, shaping adolescents’ exposure to beauty content and influencing consumer desires without requiring active engagement or intentional searching.

While participants often frame their engagement as passive exposure, these narratives may also indicate a normalised mode of algorithmically structured consumption, in which agency is partially obscured rather than absent [26,27].

### Theme 3. Parental mediation, support and gatekeeping

Parental attitudes played a significant role in shaping adolescents’ engagement with beauty practices, influencing both access to cosmetic products and the meanings attributed to beauty routines. Participants’ narratives revealed a broad spectrum of parental roles, ranging from active involvement and encouragement to cautious restriction and explicit gatekeeping. Rather than functioning as a uniform protective force, parental mediation emerged as a negotiated and relational process embedded in everyday family interactions.

This diversity of parental roles corresponds to differentiated mediation strategies, including co-use, enabling, and restrictive approaches, which shape not only access to products but also the meanings attributed to beauty practices [50].

#### Shared routines and intergenerational learning.

For some adolescents, beauty practices were integrated into routine family interactions and took the form of shared activities, mutual advice, and intergenerational knowledge exchange.

“I talk with my mum about cosmetics… how to use things. If I have something I don’t use, I give it to her. And the other way around.” (Interview 5).

“Mum said that if I don’t know how to use something, I can come to her. We often go shopping together.” (Interview 6).

These accounts indicate that beauty practices were not always perceived as individual or peer-driven activities but were sometimes embedded within familial relationships, where mothers acted as informal mentors and co-participants in cosmetic consumption.

#### Parental pride and positive reinforcement.

In several narratives, parental involvement extended beyond practical guidance to include emotional validation and positive reinforcement.

“Mum knows… she was shown and she smiled, she was proud.” (Interview 5).

“We ask each other questions [with my mum], for example, what is this brush for? Or how should this be applied? And we choose cosmetics together. Sometimes my mum suggests something to me, sometimes I suggest something to her.” (Interview 25).

Such interactions suggest that parental approval may function as a legitimising mechanism, reinforcing adolescents’ engagement in beauty practices and framing them as appropriate, shared, and socially valued activities rather than premature or problematic behaviours.

#### Everyday negotiations around spending.

Parental mediation also operated through everyday negotiations concerning spending and consumption frequency, reflecting attempts to balance acceptance with moderation.

“When I ask my mum for cosmetics… she sometimes complains like ‘cosmetics again?’, but it’s not really negative.” (Interview 22).

These narratives reveal that parental control was often subtle and situational, manifesting not through outright prohibition but through ongoing dialogue, conditional approval, and pragmatic compromise.

#### Restrictive approaches.

Not all parents encouraged early engagement with beauty practices. Some adolescents described clear parental boundaries grounded in age norms and perceived expertise.

“Mum told me not to wear makeup at this age because I’m too young… She’s more experienced and knows more about it.” (Interview 10).

Such accounts illustrate how parental authority and age-based reasoning were mobilised to delay or limit cosmetic experimentation, positioning parents as gatekeepers responsible for protecting children from practices deemed premature or inappropriate.

#### Perceptions of permissive parenting in other contexts.

Participants frequently contrasted their own experiences with what they perceived as permissive parenting elsewhere, often associating it with affluence, youthfulness of parents, or cultural differences.

“Wealthy parents… young, 20-30 years old… In America they live differently.” (Interview 21).

Some respondents expressed strong normative judgments regarding parental responsibility in the context of children’s exposure to beauty culture and online content.

“For me it’s just really strange, because when I was their [children’s] age, I would never have thought about wearing makeup or trying to highlight something using my mum’s cosmetics. I would never have imagined that at such an age children could already receive cosmetics from Sephora or other expensive drugstores and even run some kind of tutorials, when they are really still children.” (Interview 15).

“I think this is not good at all, because parents don’t know what is happening with their children and don’t know what influence the internet has on small children. I think this is really bad and may affect the future development of that child.” (Interview 16).

These statements position parental permissiveness as a moral and developmental concern, framing lack of oversight as a risk factor that enables premature adultification and uncritical engagement with digitally mediated beauty norms.

### Theme 4. Age-inappropriateness, health risks and boundary crossing

Across interviews, adolescents frequently reflected critically on what they perceived as excessive, premature, or unsuitable practices for younger children, including the use of advanced skincare ingredients, intensive multi-step routines, and heavy makeup. Importantly, these accounts were not limited to moral judgement; they also revealed mechanisms of boundary crossing, whereby age norms were simultaneously recognised and destabilised through peer comparison, retail practices, and digitally mediated consumption. Participants’ narratives thus positioned “Sephora Kids” practices at the intersection of adultification, consumer access, and perceived health-related risk.

#### Desire to appear older.

Participants often interpreted early and intensive beauty practices among younger children as motivated by a desire to appear older and to gain social acceptance. In these narratives, beauty work functioned as a strategy for performing maturity and “fitting in,” suggesting that appearance becomes a symbolic marker through which age boundaries are negotiated rather than simply followed.

“They want to look older… they have older sisters… kids compete about who is older.” (Interview 10).

“Exactly, to look older and to simply maybe feel better, to just fit in.” (Interview 25).

These accounts indicate that age-inappropriate beauty practices are frequently understood by adolescents as socially functional behaviours, used to manage peer comparison and belonging.

#### Behaviour in stores.

A recurring theme concerned behaviours in cosmetic stores, particularly the use and destruction of product testers. Participants framed these practices as both a material manifestation of boundary crossing and a consequence of consumer norms that treat products as accessible objects of experimentation without responsibility.

“Young girls use the testers, put makeup on with the, [...] the testers end up destroyed.” (Interview 14).

“It seems to me that it’s mainly children, that they follow this way of thinking that it’s for free, you can do whatever you want with it, so they destroy it. Although now, for example in drugstores like Rossmann, it’s not only the testers that are destroyed, but all cosmetics are opened and you often have to ask from the drawer for an unopened product.” (Interview 10).

These narratives link retail environments to the normalisation of early cosmetic experimentation, while also highlighting a shift from individual misuse of testers toward broader patterns of uncontrolled product handling in everyday drugstore spaces.

#### Use of adult-targeted products.

Participants frequently described younger children’s use of expensive or adult-targeted products as excessive and age-inappropriate. These accounts often combined descriptive observations with explicit normative statements about what younger children “should” or “should not” do.

“They use expensive products not intended for their age. They use too much of them.” (Interview 18).

“When I observe now, I see that quite young girls start wearing makeup. Some ten-year-olds. Quite young. They probably shouldn’t, because they are young, too young.” (Interview 27).

Such statements reflect adolescents’ awareness of age norms in beauty culture and their positioning of younger children’s practices as boundary violations, particularly when these practices involve premium products and adult routines.

#### Recognised health risks.

Perceived health risks, especially skin damage, were frequently invoked as a rationale for why certain practices were inappropriate for children and younger adolescents. Participants often framed harm as resulting not only from product strength but also from a lack of knowledge and age-related vulnerability.

“My friend was 11-12… foundation, concealer, powder… it’s unhealthy… it can damage the skin. We’re still children.” (Interview 2).

“Girls who are 12 introduce such skincare, really solid, with those acids, I heard about it. I don’t think it would be needed for them, because they can really harm themselves with those acids, if they know nothing about them [acids] and still at such an age. They shouldn’t do it, in my opinion, but it is probably a matter of the fact that they didn’t read something.” (Interview 18).

“It is better to use products that are actually adapted to teenagers’ skin. Not some 50+ creams, which […] probably in some way act differently on younger and older skin. So I think it is better to use more adapted products.” (Interview 23).

These accounts indicate that adolescents draw on a quasi-expert risk discourse to evaluate cosmetic practices, distinguishing between “adapted” and “too strong” products and emphasising that inappropriate use may result from misinformation or insufficient understanding.

#### Support for regulatory measures.

Finally, several participants explicitly supported age-based restrictions on the purchase or use of strong cosmetic products. Importantly, this support was justified through both risk-based and norm-based arguments, including references to product labelling and the notion that certain cosmetics are intended for “mature skin.”

“A ban is a good idea… young girls won’t damage their skin early. It would be better if the ban also applied in drugstores.” (Interview 5).

“It is a restriction, but it seems to me that it makes sense, because in general on the label it is written that it is for mature skin, so a person aged 12 should not buy it, because it is not for them. Is such a restriction needed? […] it is needed.” (Interview 18).

“I think that the ban is good, because people below the age of fifteen should not use such strong cosmetics, even in my opinion, below the age of eighteen they should not use such strong cosmetics.” (Interview 10).

Together, these narratives show that adolescents do not merely reproduce beauty norms but also articulate boundary-making practices of their own, including calls for formal regulation. At the same time, the emphasis on labels, age thresholds, and “strength” of products suggests that young people interpret cosmetic consumption through a framework of appropriate risk management and age-differentiated bodily needs.

These accounts indicate that adolescents do not simply reproduce dominant norms, but actively engage in boundary-making practices, combining moral judgement, risk awareness, and emerging forms of lay expertise.

## Discussion

### Early incorporation of body capital: Routinisation of embodied aesthetic practice

The findings demonstrate a strong normalisation of structured beauty routines among adolescents, suggesting that appearance is increasingly treated as a continuous and managed project rather than a spontaneous or playful practice. This aligns with Shilling’s conceptualisation of the body as a “project-in-progress” [15] and Turner’s notion of the “somatic society” [16], where the body becomes central to identity construction and social evaluation.

However, the present study extends these frameworks by showing that such bodily projectification begins earlier than previously emphasised in sociological literature. Rather than emerging in adulthood, bodily management is already routinised in early adolescence through daily skincare, product layering, and disciplined self-monitoring.

Participants’ accounts indicate that these practices are embedded in everyday normality:

“I think this is mostly my everyday routine.” (Interview 16).

The extensive routines described by participants reflect embodied forms of cultural competence that can be interpreted as emerging body capital, understood here as the early accumulation of embodied aesthetic knowledge, discipline, and classification skills. This interpretation draws on Bourdieu’s theory of embodied capital [18], while extending it to digitally mediated contexts where aesthetic knowledge is acquired through platform exposure and peer exchange.

Importantly, body capital is not treated here as a directly observable empirical entity, but as an analytical synthesis capturing the convergence of routinisation, repetition, and embodied aesthetic learning.

These practices may also be interpreted through the lens of aesthetic labour, a concept emphasising how individuals invest effort into producing an appearance that meets socially valued norms [51,52]. Although the term is typically applied to workers in aesthetic industries, the adolescents’ narratives suggest that similar expectations trickle down into everyday youth culture, transforming beauty routines into skills that generate social value. Importantly, this study shows that body capital is not only accumulated later in life or within professional contexts, but begins to take shape already in early adolescence through everyday beauty routines embedded in digital consumer cultures.

### Consumption, classed aesthetics, and adolescent consumer socialisation

The data indicate early and sophisticated forms of consumer literacy. Adolescents evaluate products in terms of price, quality, and brand legitimacy, including the use of “dupes”:

“If there’s a cheaper dupe of something expensive, why buy the expensive one? The cheaper one works the same.” (Interview 27).

This aligns with consumer socialisation theory [4,53,54], but suggests intensification under digital capitalism. Consumption is not merely learned but continuously reinforced through platform exposure, influencer culture, and algorithmic recommendation systems.

At the same time, findings indicate classed distinctions embedded in beauty consumption practices. References to premium brands, Sephora spaces, and “dupes” suggest stratified access to aesthetic goods and symbolic boundaries of taste. In this sense, aesthetic practices function as early markers of social distinction [23,24,49,55].

### Algorithmic socialisation and perceived passivity

A consistent pattern in the interviews is that adolescents do not actively seek beauty-related content; instead, they describe receiving it passively through automated recommendations. This aligns with Hepp’s concept of deep mediatization, where media infrastructures restructure everyday practices and sociocultural expectations [29,56]. As Krotz argues, mediatization transforms not only communication but the conditions under which meanings and social relationships are produced [30].

This passive mode of exposure is clearly reflected in participants’ narratives, for instance: “Popular topics just appear for me… sometimes I watch, sometimes I skip.” (Interview 21).

Interviews suggest that adolescents often perceive social media content as appearing automatically in their feeds, describing experiences of passive exposure (e.g., “it just appears”, “I watch it if it comes up”), which they interpret as shaping their engagement with beauty-related content. This aligns with scholarly discussions of algorithmic consumer culture [44] and reflects broader concerns about how digital platforms influence identity formation [57].

The interviews also highlight the aspirational dimension of influencer culture. Adolescents perceive influencers as models of a desirable lifestyle, consistent with Goffman’s dramaturgical approach in which individuals learn how to perform identity by observing others’ “front-stage” behaviours [58]. Social media further amplifies impression management, encouraging young users to document, refine, and showcase their appearance in line with platform-specific norms [59].

### Body capital, capital hierarchies and theoretical positioning

The study contributes to ongoing debates on aesthetic and cultural capital, based on adolescents’ narratives of everyday beauty practices and media use, by positioning adolescence as a formative stage in capital internalisation. In contrast to Feijoo & Vizcaíno-Verdú’s [26] concept of **s**ocial media aesthetic capital, which emphasises visible self-presentation and recognition, the present study highlights earlier processes of embodied routinisation, where aesthetic logics are incorporated into bodily practice before stable identity performance emerges, as reflected in participants’ accounts of routine exposure and self-presentation practices.

Similarly, while Kuipers [23] demonstrates how aesthetic judgement reflects emerging cultural capital, and Schneickert et al. [49] show how appearance intersects with stratification, this study shows how such logics are pre-structured through everyday routines before being expressed as evaluative judgement, grounded in how adolescents describe everyday engagement with visual and beauty-related content.

Thus, the key contribution lies in demonstrating, as suggested by the empirical material, that aesthetic and cultural capital are not only outcomes of stratification but also early-forming embodied dispositions shaped through digital consumption environments.

### Adultification and the blurring of childhood boundaries

Participants repeatedly emphasise that very young children, sometimes as young as 8–10 years old, engage in beauty routines that resemble adult practices. This corresponds to research on adultification, a process in which children are expected or encouraged to adopt behaviours, aesthetics, or responsibilities typically associated with adulthood [60].

The motivation underlying such practices was explicitly articulated by participants, who noted that younger children use cosmetics “to look older and to simply maybe feel better, to just fit in.” (Interview 25).

The desire “to look older,” frequently mentioned by participants, illustrates how consumer culture collapses traditional markers of age [34]. Xie demonstrates that adolescents increasingly internalise highly curated and unrealistic beauty standards circulating on social media, which contributes to distorted body image perceptions [35].

### The family as a site of mediation: Intergenerational consumption and conflicting norms

Parents in this study play ambivalent roles: some act as co-participants in beauty practices, others serve as gatekeepers, while still others appear permissive, facilitating access to expensive products or documenting children’s routines online. This complexity mirrors findings in studies of parental mediation of digital practices, where restrictive, enabling, and co-use frameworks coexist [50].

These roles are reflected in adolescents’ descriptions of shared routines and parental involvement, such as: “I talk with my mum about cosmetics… how to use things. If I have something I don’t use, I give it to her. And the other way around.” (Interview 5).

The intergenerational sharing of cosmetic knowledge also strengthens the argument that beauty consumption is embedded within family norms and not solely driven by peers or media.

### Health-related implications and regulatory considerations

Several participants express awareness that certain practices may be “unhealthy” or “harmful to the skin.” Nonetheless, these concerns do not deter experimentation, likely because aesthetic expectations override long-term considerations. This reflects what Widdows calls the moral imperative of beauty, where performing beauty becomes a duty linked to social belonging and self-worth [61].

Participants frequently framed these risks using quasi-expert language related to acids and skin damage, for example: “Girls who are 12 introduce such skincare, really solid, with those acids… they can really harm themselves with those acids, if they know nothing about them and still at such an age.” (Interview 18).

The interview responses endorsing age restrictions on potent skincare products echo emerging debates in public health, dermatology, and youth protection [9,62–64].

### Gender as structural condition

The strong feminisation of beauty practices (Gill & Elias) is not incidental but structurally embedded in adolescent aesthetic labour regimes [51]. While boys engage in grooming practices, these remain narrower and less intensive, reflecting gendered organisation of bodily labour.

Thus, gender is not treated as a sample limitation but as an analytic dimension of how body capital is differentially distributed and enacted.

### Conclusion of discussion

Overall, the findings demonstrate that adolescent beauty practices constitute an early site of embodied capital formation shaped by the interaction of digital infrastructures, consumer culture, and social relations. The study extends existing literature by showing that aesthetic capital logics are not only socially distributed but also developmentally internalised through routine bodily practice in early adolescence.

### Contributions, limitations and directions for further research

By foregrounding adolescents’ own interpretations of beauty practices, this study moves beyond media- or adult-centred accounts and highlights how young people actively negotiate norms of appearance, age and risk within digital consumer cultures. The findings provide an original empirical contribution to international literature by documenting the early incorporation of body-related practices into everyday adolescent routines and by situating these practices at the intersection of body sociology, consumer culture, and mediatized socialization.

Specifically, this study contributes to existing research by: (1) demonstrating how beauty routines function as early forms of embodied and aesthetic capital accumulation; (2) integrating everyday cosmetic practices into sociological analyses of youth consumption and identity work; (3) illustrating how algorithmic infrastructures shape aspirational norms and normalize age-inappropriate beauty practices; and (4) capturing adolescents’ own critical reflections on adultification, health risks, and the need for regulatory boundaries.

Several limitations should be acknowledged. First, the study is based on a non-representative sample recruited through purposive and snowball sampling, with a clear predominance of female participants. While this reflects the feminised character of beauty culture and the inclusion criterion of familiarity with the phenomenon, it limits the ability to systematically analyse gender differences. Future research should aim to include more gender-balanced samples in order to explore how boys and other gender groups engage with beauty practices and whether similar processes of early body capital incorporation occur across different populations.

Second, the study did not systematically account for detailed socio-demographic characteristics such as socioeconomic status, parental education, or household resources. Although variation in place of residence was considered, the lack of more fine-grained socio-demographic data limits the ability to assess how structural inequalities shape access to cosmetic products, consumer competencies, and parental mediation. Future studies should incorporate these variables to better understand the social differentiation of the phenomenon.

Third, the research relies exclusively on adolescents’ self-reported accounts. While this provides valuable insight into their subjective meanings and interpretations, it does not capture the perspectives of parents or caregivers, who were identified as key actors in mediating, enabling, or restricting engagement with beauty practices. Including parents in future research would allow for a more comprehensive understanding of intergenerational dynamics, perceived risks, and family-level negotiations.

Fourth, the cross-sectional design does not allow for assessment of long-term consequences of early engagement in beauty practices. In particular, it remains unclear how early routinisation of cosmetic practices may shape body image, self-esteem, or identity development over time. Longitudinal research would be particularly valuable in tracing these processes and their potential outcomes.

Finally, while participants articulated awareness of health risks and age-inappropriateness, the study did not directly examine broader psychosocial consequences of early aesthetic practices. Future research could explore more explicitly the potential risks associated with premature engagement in beauty work, including the sexualisation or adultification of appearance, increased visibility of young bodies in public and digital spaces, and the possibility of adolescents becoming objects of unwanted attention or evaluation. Such processes may have implications not only for psychological well-being but also for perceived and actual safety.

Future research would benefit in particular from comparative and longitudinal designs that examine how early engagement in beauty practices shapes embodied self-perceptions across different cultural contexts. Further studies could also explore parental mediation strategies in greater depth, including restrictive, enabling, and co-use approaches, as well as the role of platform-specific algorithms in intensifying or moderating exposure to appearance-focused content. Additionally, integrating perspectives from parents, educators, and practitioners would allow for a more comprehensive understanding of the social and developmental implications of the “Sephora Kids” phenomenon.

## Conclusion

This study examined adolescents’ beauty practices as everyday, socially embedded activities shaped by digital media, consumer culture, and intergenerational dynamics. The findings show that engagement in skincare and cosmetic routines is highly normalised and often organised as structured, habitual practices that resemble adult regimes. Social media platforms—particularly through algorithmic exposure—play a central role in shaping beauty knowledge, aspirational norms, and consumer desires, while peer and family contexts provide additional frameworks for validation, negotiation, and control.

By foregrounding adolescents’ own perspectives, the study demonstrates that young people are not passive recipients of beauty norms but active interpreters who simultaneously reproduce, negotiate, and sometimes contest age-related boundaries and risk discourses. At the same time, the routinisation of beauty practices and the development of consumer and aesthetic competencies can be interpreted as early stages in the incorporation of bodily resources that may function as forms of body capital.

Importantly, the findings point to a broader transformation in the timing and social organisation of appearance-related practices, where activities traditionally associated with later life stages are increasingly embedded in early adolescence. This shift raises important questions about the changing nature of childhood, the role of digital infrastructures in shaping identity formation, and the growing significance of the body as a site of investment, evaluation, and social differentiation.

Taken together, the study contributes to ongoing debates on youth, mediatization, and the commercialization of the body, while also highlighting the need for further research into the long-term social, psychological, and developmental implications of early engagement in beauty culture.

## Supporting information

S1 File Interview guide used in the study. (DOCX)

S1 Table Characteristics of the interview participants. (DOCX)
