# Supplementary material for: Early incorporation of body capital in adolescent beauty culture: A qualitative study of the ‘Sephora Kids’ phenomenon
Source: PLoS One. 2026 Sep 16;21(9):e0356694. doi: 10.1371/journal.pone.0356694 (PMC13580945; doi:10.1371/journal.pone.0356694)
Supplement: Supplementary file 1 [file pone.0356694.s001.docx]

# **S1 File. Semi-Structured Interview Guide**

# **(In-Depth Interviews with Adolescents)**

## **Overview**

This study employed a semi-structured in-depth interview (IDI) guide designed to explore adolescents’ skincare practices, beauty-related consumption, and perceptions of the “Sephora Kids” phenomenon.
The guide was organised around thematic blocks corresponding to the study’s research objectives. All core questions were addressed in each interview, while follow-up questions and probes were used flexibly to explore participants’ experiences and meanings in greater depth. Not all follow-up questions were asked in every interview, in line with the principles of qualitative interviewing.

## **1. Everyday skincare and beauty routines**

- Can you describe your daily skincare or makeup routine?
- What products do you usually use?
- When did you start using skincare or makeup products?
- Has your routine changed over time? If so, how?

Optional probes:
– What does this routine mean to you?
– How much time do you usually spend on it?
– Are there days when you skip it? Why?

## **2. Sources of beauty knowledge**

- Where do you usually get information about cosmetics or skincare?
- Who or what influences your choices the most?
- Do you rely more on online sources or people you know personally?

Optional probes:
– Are there specific influencers, accounts, or platforms you follow?
– Do you ever compare different opinions or recommendations before trying a product?

## **3. Social media and beauty-related content**

- Do you watch beauty-related content on social media platforms?
- What kind of beauty content do you see most often?
- How does this content make you feel about your own appearance or skincare routines?

Optional probes:
– Have you ever tried a product because you saw it online?
– Do you trust influencers’ recommendations? Why or why not?
– Do you notice trends or “viral” products?

## **4. Family influence and parental mediation**

- Do you talk with your parents about cosmetics or skincare?
- What do your parents think about the products you use?
- Do they influence or limit your choices in any way?

Optional probes:
– Do your parents help you choose products or check ingredients?
– Have you ever disagreed with them about cosmetics or makeup use?

## **5. Beauty, age, and social norms**

- At what age do you think it is appropriate to start wearing makeup or using skincare products?
- How do you decide whether a product is “for children” or “for adults”?
- Do you think age matters when it comes to beauty routines?

Optional probes:
– Have your views on this changed over time?
– Do your peers have similar or different opinions?

## **6. Perceptions of the “Sephora Kids” phenomenon**

- How would you describe the “Sephora Kids” phenomenon?
- What do you think about children using advanced or premium skincare products?
- How is this phenomenon discussed among your peers or online?

Optional probes:
– Do you see yourself as part of this phenomenon? Why or why not?
– How do people react to it on social media?

## **7. Health risks and age-related boundaries**

- Do you think some cosmetic products are not suitable for younger children?
- Have you ever worried that a product might be harmful?
- Where do you get information about product safety?

Optional probes:
– Do influencers talk about risks or side effects?
– Do you check ingredients or warnings before using products?

## **8. Consumption practices and access to cosmetics**

- Where do you usually get your cosmetics?
- How do you pay for them?
- What factors matter most when choosing a product (e.g., brand, price, recommendations)?

Optional probes:
– Do you ever buy products impulsively?
– Do you receive cosmetics as gifts?

## **9. Closing reflections**

- Is there anything else you would like to add about skincare, makeup, or beauty practices among young people?
- Is there something adults should better understand about this topic?

## **Sociodemographic information**

At the end of the interview, participants were asked about:

- age,
- gender identity,
- place of residence,
- social media platforms used,
- typical places of purchasing cosmetics,
- use of premium cosmetic products.
