# Supplementary material for: Early incorporation of body capital in adolescent beauty culture: A qualitative study of the ‘Sephora Kids’ phenomenon
Source: PLoS One. 2026 Sep 16;21(9):e0356694. doi: 10.1371/journal.pone.0356694 (PMC13580945; doi:10.1371/journal.pone.0356694)
Supplement: Supplementary file 2 [file pone.0356694.s002.docx]

# S1 Table. Characteristics of the interview participants

| Interview ID | Gender | Place of residence (category) | Social media platforms used |
| --- | --- | --- | --- |
| 1 | Female | Town <50,000 | YouTube, Facebook |
| 2 | Female | Town <50,000 | TikTok, YouTube |
| 3 | Female | Town <50,000 | Instagram, TikTok, YouTube, Messenger |
| 4 | Female | Town <50,000 | Instagram, TikTok, Facebook, Snapchat, YouTube, Pinterest |
| 5 | Female | Rural | Instagram, Snapchat, TikTok, BeReal |
| 6 | Female | City >500,000 | Instagram, Facebook, Snapchat, TikTok, YouTube, BeReal |
| 7 | Male | City >500,000 | TikTok, Facebook, Instagram, YouTube |
| 8 | Male | City >500,000 | YouTube, TikTok, Instagram, Facebook |
| 9 | Female | City 100,000–300,000 | Instagram, TikTok, YouTube |
| 10 | Female | City 50,000–100,000 | YouTube, WhatsApp |
| 11 | Female | City 100,000–300,000 | Instagram, TikTok, Snapchat, YouTube |
| 12 | Female | Town <50,000 | Instagram, TikTok, Facebook, Snapchat, YouTube |
| 13 | Female | Rural | Instagram, Facebook, TikTok, YouTube |
| 14 | Female | City 300,000–500,000 | Facebook, YouTube, Pinterest |
| 15 | Female | City 100,000–300,000 | Instagram, TikTok, Snapchat |
| 16 | Female | City 100,000–300,000 | Instagram, TikTok, Snapchat, YouTube |
| 17 | Female | Rural | Instagram, TikTok, Snapchat, YouTube, WhatsApp, BeReal |
| 18 | Female | Town <50,000 | Instagram, YouTube |
| 19 | Female | Town <50,000 | Instagram, TikTok, Facebook, Snapchat, YouTube |
| 20 | Female | Rural | TikTok, YouTube, Instagram |
| 21 | Female | Rural | Instagram, TikTok, Facebook, Snapchat, YouTube |
| 22 | Female | Rural | Instagram, TikTok, YouTube |
| 23 | Female | Rural | TikTok, Instagram, YouTube, Facebook |
| 24 | Female | City 100,000–300,000 | Instagram, Snapchat, YouTube |
| 25 | Female | City >500,000 | Instagram, TikTok |
| 26 | Female | City 100,000–300,000 | TikTok |
| 27 | Female | Rural | Instagram, TikTok, Facebook, Snapchat, YouTube |
| 28 | Female | City 300,000–500,000 | Instagram, TikTok, Facebook, YouTube |
| 29 | Female | City 300,000–500,000 | Instagram, TikTok, Facebook |
| 30 | Male | City 300,000–500,000 | Facebook, YouTube, Instagram |
